# A Comprehensive Review of Severe Acute Respiratory Syndrome Coronavirus 2

**DOI:** 10.7759/cureus.7943

**Published:** 2020-05-03

**Authors:** Woong Kee Baek, Soo-Yeon Sohn, Ahmed Mahgoub, Robert Hage

**Affiliations:** 1 Anatomical Sciences, St. George's University School of Medicine, St. George, GRD; 2 Dentistry, Virginia Commonwealth University School of Dentistry, Richmond, USA; 3 Otolaryngology, St. George's University School of Medicine, St. George, GRD

**Keywords:** sars-cov-2 (severe acute respiratory syndrome coronavirus -2), novel coronavirus, coronavirus disease (covid-19)

## Abstract

Severe acute respiratory syndrome-coronavirus 2 (SARS-CoV-2), the virus strain that causes coronavirus disease 2019 (COVID-19), was first identified in Wuhan, China in December 2019. It spread to several countries across continents and infected more than one million people within three months. While there is no consensus on the treatment of the disease yet, understanding the virus and its transmission is a cardinal priority. SARS-CoV-2 can be transmitted through bodily fluid. Upon inoculation, the surface enzyme angiotensin-converting enzyme 2 (ACE2) acts as a receptor protein for viral entry. The mean incubation period is 5.1 days, and infected individuals can exhibit a variety of symptoms from fever, cough, dyspnea, and respiratory failure to even multiorgan failure. Given the current situation, it is of paramount importance to understand the virus as thoroughly as possible. In this review, we discuss the background, epidemiology, possible pathophysiology, clinical presentation, and diagnostic studies related to SARS-CoV-2 infection. We also elaborate on the current research and evidence on treatment options and vaccine development based on the literature.

## Introduction and background

Severe acute respiratory syndrome-coronavirus 2 (SARS-CoV-2), the virus strain that causes coronavirus disease 2019 (COVID-19), has been reported in several countries across continents since it was first identified in December 2019. It infected nearly 1.7 million people within four months [1]. The virus first appeared in a cluster of atypical pneumonia cases in Wuhan, China, possibly related to a seafood market. On January 12, 2020, the virus was initially named the 2019-novel coronavirus (2019-nCov) by the World Health Organization (WHO). From the discovery of the initial case in Wuhan, it rapidly spread across China and many other countries, eventually affecting several continents. On January 30, 2020, the WHO officially declared the COVID-19 epidemic a public health emergency of international concern. On February 11, 2020, the disease was officially named COVID-19 by the WHO and the virus was named SARS-CoV-2 by the Coronavirus Study Group of the International Committee [2].

SARS-CoV-2 is a 125nm-diameter, spherical-shaped β-coronavirus. Other zoonotic β-coronaviruses subsets are known to cause Middle East Respiratory Syndrome (MERS) and Severe Acute Respiratory Syndrome (SARS). The nucleocapsid encloses a positive-sense single-stranded RNA virus. Coronaviruses have four main structural proteins: spike protein (S), membrane protein (M), envelope protein (E), and nucleocapsid (N), while β-coronavirus contains a fifth structural protein, hemagglutinin-esterase (HE), which is analogous to hemagglutinin [3].

## Review

Epidemiology

The median incubation period of the infection is 5.1 days, with 97.5% of the population showing symptoms within 12 days of exposure [4]. The basic reproduction number (R_0_), which is the indicator of the transmissibility of the disease, was derived from the mean of 12 studies between December 2019 to January 2020 and is 3.28 with a median of 2.79 (range: 1.4-6.49) [5]. However, this number is still evolving with a most recent study from the Centers for Disease Control and Prevention reporting a median R_0_ as high as 5.7 (95% CI: 3.8-8.9) based on the assumed serial interval (six to nine days) [6]. The case fatality rate (CFR) of SARS-CoV-2 infection ranges widely between countries, from 7.2% in Italy to 0.7% in South Korea, due to numerous factors [7,8]. However, these numbers are subject to change in the future as, during an epidemic, studies only capture a cross-sectional/temporal representation at the time of assessment. More reflective values can be obtained once the epidemic is over. As the number of disease cases changes rapidly, conducting a precise epidemiological study is challenging.

Genetics

The complete reference genome sequence data of SARS-CoV-2 was made available in December 2019, and the sequence data and variations have been updated at GenBank [9]. SARS-CoV-2 is 96.2% identical to a bat CoV RaTG13 and shares 79.5% of its identity with SARS-CoV, suggesting the bat as the most likely natural host of the virus. However, it is suspected that the zoonotic infection took place via an unknown intermediate host [2].

Pathogenesis

Viral Attachment and Entry

The molecular protein responsible for the entry of SARS-CoV-2 into human host cells is angiotensin-converting enzyme 2 (ACE2) [10]. This protein is widely found in different organs such as the lung, kidney, heart, and endothelial tissue. The main functions of ACE2 are the downregulation of the renin-angiotensin-system (RAS), balancing the overdrive of RAS mediated response and the renal, gastrointestinal absorption of amino acids [11]. It also acts as a means of clathrin-mediated internalization of viruses such as SARS coronavirus [11]. Recent studies have revealed that ACE2 interacts with transmembrane protease, serine 2 (TMPRSS2), which is responsible for the S protein activation of SARS-CoV-2, just as in SARS coronavirus [3,10,12]. S protein of the viral structure interacts with surface ACE2 enzyme, which leads to the internalization of the viral material. As the viral RNA is released into the host cytoplasm, the viral translation process takes place using the host cellular machinery [10].

Protein Synthesis and Viral Replication

The viral replicase gene of the single-stranded RNA is translated to produce key proteins such as RNA-dependent RNA polymerase (RdRp), RNA helicase, and other non-structural proteins, which will serve as replicase-transcriptase complex (RTC) [3,13]. Further to RTC translation, subsequent viral RNA synthesis takes place using RdRp through a complementary strand as an intermediate. The translation products of the viral sub-genomic RNA are further processed, and this results in a series of viral structural S, E, N, and M proteins. They are subsequently used in the assembly process with viral genomic RNA, which is then released [3].

G*eneral Viral Infection and Immune Response*

Upon viral infection, the immune system responds mainly in three ways. At the early phase of the infection, an innate immune response deploys myeloid lineage cells, natural killer (NK) cells, and epithelial cells, which, upon activation, will secrete proinflammatory cytokines and chemotactic factors. These include but not limited to interleukins (IL) -1, -6, -8, -12, -15, -18, tumor necrosis factors (TNF), granulocyte-macrophage (GM)-, granulocyte (G)-, macrophage colony-stimulating factors (M-CSF), and interferon-gamma (IFN-ɣ) 1, 2, 3. These cytokines act to induce specific cells, including NK and dendritic cells [14]. Dendritic cells of the respiratory system can be directly and indirectly activated upon viral respiratory infection. Epithelial cells release IL-1 and then GM-CSF to recruit dendritic cells and secrete IL-33 to support paracrine regeneration [14]. In the lung, type II pneumocytes (T2P) also participate during the immune response: T2P produces surfactant, which is composed of phospholipids (mainly phosphatidylcholine and phosphatidylglycerol), lipid (cholesterol), and surfactant protein A, B, C, D. The surfactant A and D pair with viruses to promote the action of macrophages [15]. An effective early phase defense mechanism depends on phagocytic cell interaction with antigens directly using pattern recognition receptors (PRR) and pathogen-associated molecular patterns (PAMP) or indirectly via opsonins. The adaptive immune response, especially the arm of T cell maturation, is in majority through an increase in IL-2, followed later by a CD4+Th cell response and CD8+T-cell-mediated cytotoxic response. Through the process of autophagy, harmful and damaged cellular components are metabolically degraded [16]. Autophagy also plays an important role in modulating the innate and adaptive responses during a viral infection as any pathogenetic steps of viral infection from its entry, fusion, and to the interference of cellular homeostasis can disrupt the immune-protective mechanism [16].

SARS-CoV-2 Infection and Its Possible Pathophysiology and the Immune Response

A similar early innate response is seen in SARS-CoV-2-infected patients who present with initial cardinal symptoms such as fever and malaise. Yet, their clinical sequelae and the response to the treatment are different from any respiratory diseases known today. The increase in the body temperature seems to be due to the production of proinflammatory cytokines such as IL-1, -6, -8, -10, -12, and TNFs from first responders such as monocytes, damaged local endothelial cells, and neutrophils. While macrophages promote the local inflammatory response with the release of chemotactic factors, the paracrine response of the endothelial cells and pneumocytes enhances the expression of adhesion molecules such as intercellular adhesion molecule 1 (ICAM-1) on T2P. E-selectin and vascular cell adhesion molecule (VCAM) facilitate the migration of white blood cells.

It is suspected that the acute respiratory distress syndrome (ARDS)-like picture in SARS-CoV-2-infected patients is precipitated and worsened by the excess monocytes in response to GM-CSF, which is released by rapidly activated CD4+T-cell lineage [17]. The progression to ARDS-like presentation is driven by three main factors: the local inflammatory response by neutrophils inducing local damage; the releasing of proteinaceous cellular debris in the alveolar space; and locally-acting mediators such as endothelin-1, phospholipase A-2, and angiotensin-2 making the local vessel more permeable. The damage to type I pneumocytes impedes local gas exchange while damage to type II pneumocytes results in decreased surfactant production. The surfactant has an important function in reducing the surface tension of alveoli. As more T2P are lost, the surface tension of the alveoli increases, which leads to the collapse of the alveolar cavity. Furthermore, the alveolar cavity is filled with the proteinaceous cellular debris from the inflammatory reactions and the exudate from the increase in vascular permeability, which translates into the pulmonary edema-like picture in patients. The infection spreads to the adjacent alveoli through the pores of Kohn, infecting adjacent alveolar cavities. These events explain the progression of lung involvement, dyspnea, and shortness of breath. Yet, ARDS-like presentation with SARS-CoV-2 infection is atypical as the patients who fit into the Berlin Definition do not show similar lung compliance and shunt fraction as a typical ARDS patient [18].

Cytokine Storm

SARS-CoV-2-infected patients have shown a rise of cytokines during their disease course, notably, IL-2, -4, -6, -7, -10, TNF-α, G-CSF, interferon-gamma-inducible protein (IP), monocyte chemoattractant protein 1 (MCP1), and macrophage inflammatory protein 1 alpha (MIP1A), between day 3-10 of disease onset, which corresponded to the disease severity of the patients [17,19,20]. Patients with mild symptoms presented with an elevation of cytokines within the reference ranges, while severe patients showed markedly elevated levels [19]. As for the IFN-ɣ, there are mixed findings [19,21]. Zhou et al. have considered the high IFN-ɣ and GM-CSF by Th1 cells and GM-CSF by CD8+T-cells as the possible cause of the hyper-inflammatory reaction, as NK cells and B-cells were not found to relate with these features [21]. Given that monocytes respond to GM-CSF as expected, SARS-CoV-2-infected patients had higher CD14+CD16+ monocytes than normal healthy individuals [17,21]. These monocytes promote the secretion of GM-CSF and IL-6, both of which are surged in severely ill patients, overfeeding the inflammatory response, and suggesting the relationship among GM-CSF, IL-6, monocytes, and Th1 cells, which explains the cytokine storm [17,20,21].

A recent study of SARS-CoV has shown that T-cell dysfunction along with the decrease in type I IFN is related to T-cell exhaustion [22]. Yet, the relationship between the clinical severity of SARS-CoV-2-infected patients and T-cell exhaustion is still unknown. Mehta P et al. have reported that the cytokine profile and the trend of the inflammatory markers of SARS-CoV-2-infected patients present similarly to the secondary hemophagocytic lymphohistiocytosis (sHLH), whose severe clinical presentation is related to the cytokine storm [23]. Further study is needed to verify if the severe presentation and poor prognosis are related to any of these aforementioned findings.

Clinical presentation

A spectrum of symptoms is observed in SARS-CoV-2-infected patients. The degree of the severity of the disease experienced by a diverse population is very different as well. While the majority of patients experience cardinal and respiratory symptoms, a minority of the population also presents with gastrointestinal symptoms such as diarrhea, nausea, and vomiting. The most commonly reported symptoms are fever, dry cough, and fatigue followed by myalgia, chest tightness/pain, sore throat, shortness of breath, dyspnea, and rhinorrhea (Tables 1, 2) [19,24-27].

**Table 1:** Clinical presentation of SARS-CoV-2-infected patients, reproduced and modified from Study 1* SARS-CoV-2: severe acute respiratory syndrome coronavirus 2; ICU: intensive care unit; Non-ICU: non-intensive care unit *Data reproduced and modified from Wang et al.’s research [25]

Parameter from Study 1*	Number of patients*	ICU*	Non-ICU*	Patients with severe disease, %*	Patients with symptoms, %
Total patients	138	36	102	74%	
Median age, years	56	66	51		
Fever	136	36	100	74%	99%
Fatigue	96	29	67	70%	70%
Dry cough	82	21	61	74%	59%
Anorexia	55	24	31	56%	40%
Myalgia	48	12	36	75%	35%
Dyspnea	43	23	20	47%	31%
Expectoration	37	8	29	78%	27%
Sore throat	24	12	12	50%	17%
Diarrhea	14	6	8	57%	10%
Nausea	14	4	10	71%	10%
Headache	9	3	6	67%	7%
Vomiting	5	3	2	40%	4%
Abdominal pain	3	3	0	0%	2%

**Table 2:** Clinical presentation of SARS-CoV-2-infected patients, reproduced and modified from Study 2* SARS-CoV-2: severe acute respiratory syndrome coronavirus 2; ARDS: acute respiratory distress syndrome; non-ARDS: non-acute respiratory distress syndrome Data reproduced and modified from Liu et al.’s research [24]

Parameters from Study 2*	Number of patients*	Non-ARDS*	ARDS*	ARDS, %*	Patients with symptoms, %
Total patients	109	56	53	49%	
Median age, years	55	49	61		
Fever	90	48	42	47%	83%
Dry cough	67	36	31	46%	61%
Fatigue	62	26	36	58%	57%
Diarrhea	12	6	6	50%	11%

Liu et al. have reported that the severity of atypical pneumonia correlated with an increase in different criteria such as Sequential Organ Failure Assessment (SOFA), Acute Physiologic Assessment and Chronic Health Evaluation II (APACHE II), and CURB 65 (the acronym for criterion assessing the risk of patients presenting with respiratory diseases) [24]. Wang et al.’s study found a similar tendency among SARS-CoV-2-infected patients [25]. The progression of atypical pneumonia and respiratory failure were reported as sequelae of this disease with other severe complications such as multiple organ failure, sepsis, and death. The correlation may suggest poor prognosis; however, a meta-analysis is required to support this. The level of IL-6 is also suggested as being associated with a severe presentation of respiratory symptoms, possibly related to the cytokine storm [24,28]. In addition, Onder et al. have shown a significant case fatality in relation to the increase in the age of patients [7].

On the other side of the spectrum, although uncommon, neurological symptoms have been reported, suggesting neurologic deficits can be associated with SARS-CoV-2 infection. Zhao et al. have reported a case of a possible association with Guillain-Barré syndrome (GBS), where a 61-year-old woman acutely developed symmetric lower extremity weakness eight days before the onset of fever and respiratory symptoms [29]. Another case, a post-infectious acute hemorrhagic necrotizing encephalopathy, was reported in a patient who had a three-day history of altered mental status with cough and fever [30]. However, it is difficult to conclude if SARS-CoV-2 infection is associated with these specific diagnoses added based simply on two individual case reports. More data and studies are needed to elucidate this relationship and risk population.

Diagnostic studies

Clinical Diagnosis

There is no identified pathognomonic clinical presentation of SARS-CoV-2 infection. At the initial phase of the epidemic, high clinical suspicion was related to the presence of fever, malaise, respiratory or gastrointestinal symptoms along with a recent travel history to Wuhan, China, or physical contact with people at high risk, which were used to determine if further investigations were indicated. As the pandemic progressed and the chains of spreads took root within many local communities, more emphasis was put on the importance of diagnostic studies rather than clinical presentation. Wang et al. have stratified the SARS-CoV-2-infected patients’ presentation according to the symptom severity (Table 3) [31].

**Table 3:** Classification of symptom severity of SARS-CoV-2 infection using the reported criteria SARS-CoV-2: severe acute respiratory syndrome coronavirus 2; PaO2: partial pressure of oxygen; FiO2: fraction of inspired oxygen

Symptom severity	Description
Mild	Mild symptoms including fever, fatigue + absence of radiographic feature + absence of pneumonia-like symptom [31]
Moderate	Fever + respiratory symptoms + presence of radiographic features [31]
Severe	Dyspnea, respiration rate of >30/min or O_2_ saturation of <93% or P_a_O_2_/F_i_O_2 _of <300 mmHg [31]
Critical	Respiratory failure or septic shock or multiple organ failure [31]

Laboratory Diagnosis

Many non-specific inflammatory markers, including C-reactive protein (CRP), erythrocyte sedimentation rate (ESR), and ferritin are elevated in SARS-CoV-2-infected patients [25,31]. Other laboratory findings include elevated procalcitonin, decreased total lymphocytes count, prolonged prothrombin time (PTT), elevated D-dimer, alanine transaminase (ALT), aspartate transaminase (AST), blood urea nitrogen (BUN), creatinine (Cr), lactate dehydrogenase (LDH), and creatine kinase (CK), which were generally found to be more pronounced in patients admitted to the intensive care unit (ICU) [25]. Patients in the ICU had high levels of cytokines such as IL -2, -7, -10, TNF- α, G-CSF, and MIP1A [20]. Critically ill patients and non-survivors had high levels of neutrophils, D-dimer, ferritin, BUN, and Cr, suggesting a possible pathological etiology related to a poor prognosis [25].

Laboratory tests for diagnosing SARS-CoV-2 include nucleic acid amplification tests (NAAT) such as real-time reverse-transcriptase polymerase chain reaction (RT-PCR), serological testing such as enzyme-linked immunosorbent assay (ELISA), and viral sequencing [32]. Each diagnostic method has its own advantages and disadvantages. Currently, RT-PCR is most favored for its practicality, time efficiency, and cost factor [31].

Imaging Studies

Radiographic features are usually absent in mild cases of SARS-CoV-2 infection. Common early-stage CT findings up to day four after symptom onset have shown numerous focal ‘ground-glass opacities’ in the majority of patients [33]. Between day 5-13, lung involvement worsened with increasing consolidation in several lobes of both lungs. These pulmonary pathologies started to improve after day 14 but a complete resolution was hardly achieved even after 25 days [33]. Other manifestations included vascular thickening, halo- or air bronchogram sign, and pleural effusion, although these have been rarely encountered [31,33]. Ultrasound (US) images have supported CT findings confirming thickened pleural lines and the location of lesions [31,34]. US shows B lines in the early stage and in mild infections with the alveolar interstitial syndrome as the disease progresses [34].

Current research and evidence

Treatment Options

There is no consensus yet on how to treat SARS-CoV-2-infected patients who present with a wide spectrum of clinical symptoms and severity. The current use of hydroxychloroquine is based on the knowledge of minimizing the viral entry into the host cell and impeding viral replication by disrupting the assembly and release of the virus [35]. Mukherjee et al. have reported a favorable outcome in a 49-year-old patient who had multifocal atypical pneumonia and treated with hydroxychloroquine [26]. A recent non-randomized clinical trial in France, without a control group, has shown a positive result of the use of hydroxychloroquine with azithromycin in SARS-CoV-2-infected patients, leading to a faster resolution of symptoms in the majority of patients [36]. An adenosine analog, remdesivir (GS5734), acts as a nucleotide analog that halts the transcription process of viral RNA [37]. Holshue et al. have reported successful treatment with remdesivir of the first SARS-CoV-2-infected patient in the United States [27]. Wang et al. have reported positive effects of remdesivir and chloroquine in a study using infected cells [38]. However, not all studies are in favor of the use of these drugs. A small pilot study of 30 patients in China found no statistically significant difference in the remission of the disease between the hydroxychloroquine treatment group and the control [39]. Lopinavir and ritonavir, novel protease inhibitors that are used to treat HIV infection, are other medications proposed to treat SARS-CoV-2 infection. Cao et al. found through a randomized, controlled trial of 199 patients that the lopinavir-ritonavir treated group did not show a statistically significant difference in the mortality, hazard ratio, and viral load as compared to patients who had standard care [40]. Given that many antiviral treatments are related to severe side effect profiles, a precautious determination of the right dosage and appropriate targets should be determined through clinical trials.

Vaccine Development

Structural proteins such as glycoprotein or spike protein are important targets of antiviral vaccine development. Given that the genomic sequence of the amino acid can be used for homology remodeling, it may, theoretically, assist in identifying molecules with possible antiviral properties [41]. Yet, the high potential of spontaneous mutation associated with the genetic properties of the coronavirus, such as a high recombination ability and the presence of splicing sites and more than 40 RNA modification sites may pose challenges to this as it can promote changes of target tissue specificity and antiviral sensitivity [42].

Protease Inhibitor Targeting S Spike Protein

In addition to the presence of ACE2, an important serine protease, TMPRSS2 is used in S protein priming. This process is shown to play an important role in viral entry by mediating viral envelope fusion with the cell membrane into the host cell and, hence, is related to infectivity [10]. For this reason, Hoffmann et al. proposed a serine TMPRSS2 protease inhibitor, camostat mesylate, for its potential benefit [10]. However, this needs further studies as SARS, another subset of coronavirus, can enter host cells via an alternative pathway using endosome when the key proteases are lacking [12].

Prevention

With the absence of a treatment of choice, preventive measures are the most effective way to minimize the spread of infection. The infection primarily spreads via respiratory secretions and droplets, direct contact by touching contaminated surfaces, and/or indirectly via fomites. Transmission can occur from both symptomatic and asymptomatic patients, before symptom onset. Infected droplets can spread 1-2 m and can deposit on surfaces where they remain for a period of time. The degeneration of infectious material is accelerated by common disinfectants like sodium hypochlorite and hydrogen peroxide. Some studies have also indicated this virus to be present in stool and contaminated water supplies, suggesting possible transmission via the fecal-oral route [43]. Currently, preventive measures include the appropriate use of personal protective equipment (PPE), social distancing, and hand-washing.

Discussion

It is hard to predict where this infection can lead. With a relatively high number of reproducibility, a long incubation period, and possible transmissibility even during an asymptomatic period, SARS-CoV-2 has infected over 1.69 million people worldwide as of April 12, 2020, less than four months after its first identification at the end of December 2019 [1]. Being an enveloped positive-sense single-stranded RNA virus, its mutation rate is higher than the DNA counterparts that use host replication machinery. As the number of infected people increases, different selection pressures can derive an emergence of variant strain and/or the survival of the fittest. Tang et al. have reported two different strains of SARS-Cov-2: L and S subtypes, with S subtype being the ancestral one. However, the progenic L subtype presents higher transmissibility [44]. In addition, a recent Chinese case report has described a patient shedding virus for 49 days, which is unusually longer as compared to the median duration of 20 days [45]. This suggests that the variation of genotype is still emerging and that mutations can occur anytime in the future.

The pathophysiology behind the peripheral leukopenia in severe patients is not fully understood. Many viruses are known to interfere with T-cell activation, maturation, or programmed cell death. Recent research has shown that MERS-CoV, another coronavirus, acts to promote the intrinsic and extrinsic pathway of T-cell apoptosis, thereby decreasing the T-cell population [46]. CD4+T-cells are crucial in promoting humoral immunity and B-cell activation. The loss of Th1, Th2, and Th17 populations implies a decrease in the efficacy of mounting the adaptive immune response. Interestingly, the patient population who had severe symptomatic presentation showed more pronounced leukopenia, especially during the early phase (four to six days after disease onset) of the disease course, with an increase in cytokines (Original research article: Liu J, Li S, Liu J. et al. Longitudinal Characteristics of Lymphocyte Responses and Cytokine Profiles in the Peripheral Blood of SARS-CoV-2 Infected Patients; 2020). Zheng et al. have proposed that this may be due to the functional exhaustion of T-cells [47]. And Zhou et al. have identified the high expression of both T-cell immunoglobulin and mucin domain 3 (Tim-3) and programmed cell death-1 (PD-1) to support this idea [21]. More studies are needed in the future to elucidate the pathogenesis behind the peripheral cytopenia and immune dysregulation.

A minority of patients on remission presented back with clinical symptoms of the disease relapse or reactivation between 4-17 days after discharge [48]. Chen et al. have reported a case of recurrence of viral RNA after two consecutive days of negative tests [49]. Ye et al. have reported a rate of reactivation as high as 9% in their study, but their patients’ clinical presentation was not as critical as the initial infection [48]. The reactivations were confirmed using positive RT-PCR. The study suggested that the reactivation is associated with the degree of the immune status of the patients, the genotypic factor of the virus, and the baseline viral load. It also pointed out that all patients with reactivations were treated with antiviral medication such as oseltamivir or arbidol [48]. Although the clinical presentation and sequelae are not as severe in the patients with reactivation, there is still a risk of droplet-transmission to others upon discharge, and hence can pose a public health concern if ignored. It will be necessary to educate the patients on the possibility of reactivation and encourage a 14-day self-isolation to minimize contact with others while reporting to the appropriate healthcare agency if symptoms recur.

## Conclusions

SARS-CoV-2 infection or COVID-19 is a highly transmissible novel zoonotic infection with high case fatality rates. The transmission of infection from both asymptomatic and symptomatic patients and its relatively long incubation period make it even harder to control its spread. Unfortunately, there is no consensus on how to treat this infectious disease yet. More studies and data are needed. A good understanding of the disease transmission, its genetics, pathophysiology, clinical presentation, prognostic factors, diagnostic studies, and preventive measures are of prime importance in making clinical decisions and designing research projects for best treatment while responding to minimize the threat to people.

## References

[1] (2020). WHO: Coronavirus disease 2019 (COVID-19): situation report - 83. https://www.who.int/docs/default-source/coronaviruse/situation-reports/20200412-sitrep-83-covid-19.pdf?sfvrsn=697ce98d_4.

[2] Guo YR, Cao QD, Hong ZS (2020). The origin, transmission and clinical therapies on coronavirus disease 2019 (COVID-19) outbreak - an update on the status. Mil Med Res.

[3] Fehr AR, Perlman S (2015). Coronaviruses: an overview of their replication and pathogenesis. Methods Mol Biol.

[4] Lauer SA, Grantz KH, Bi Q (2020). The incubation period of coronavirus disease 2019 (COVID-19) from publicly reported confirmed cases: estimation and application (Epub ahead of print). Ann Intern Med.

[5] Liu Y, Gayle AA, Wilder-Smith A, Rocklöv J (2020). The reproductive number of COVID-19 is higher compared to SARS coronavirus. J Travel Med.

[6] (2020). CDC: high contagiousness and rapid spread of severe acute respiratory syndrome coronavirus 2. https://wwwnc.cdc.gov/eid/article/26/7/20-0282_article.

[7] Onder G, Rezza G, Brusaferro S (2020). Case-fatality rate and characteristics of patients dying in relation to COVID-19 in Italy (Epub ahead of print). JAMA.

[8] Shim E, Tariq A, Choi W, Lee Y, Chowell G (2020). Transmission potential and severity of COVID-19 in South Korea (Epub ahead of print). Int J Infect Dis.

[9] (2020). NCBI: SARS-CoV-2 (Severe acute respiratory syndrome coronavirus 2) sequences. https://www.ncbi.nlm.nih.gov/genbank/sars-cov-2-seqs/.

[10] Hoffmann M, Kleine-Weber H, Schroeder S (2020). SARS-CoV-2 cell entry depends on ACE2 and TMPRSS2 and is blocked by a clinically proven protease inhibitor. Cell.

[11] Kuba K, Imai Y, Ohto-Nakanishi T, Penniger JM (2010). Trilogy of ACE2: a peptidase in the renin-angiotensin system, a SARS receptor, and a partner for amino acid transporters. Pharmacol Ther.

[12] Matsuyama S, Nagata N, Shirato K, Kawase M, Takeda M, Taguchi F (2010). Efficient activation of the severe acute respiratory syndrome coronavirus spike protein by the transmembrane protease TMPRSS2. J Virol.

[13] Maier HJ, Britton P (2020). Involvement of autophagy in coronavirus replication. Viruses.

[14] Braciale TJ, Hahn YS (2013). Immunity to viruses. Immunol Rev.

[15] Martin TR, Frevert CW (2005). Innate immunity in the lungs. Proc Am Thorac Soc.

[16] Perot BP, Ingersoll MA, Albert ML (2013). The impact of macroautophagy on CD8(+) T-cell-mediated antiviral immunity. Immunol Rev.

[17] Zhou Y, Fu B, Zheng X (2020). Aberrant pathogenic GM-CSF+ T cells and inflammatory CD14+CD16+ monocytes in severe pulmonary syndrome patients of a new coronavirus (IN PRESS). bioRxiv.

[18] Ranieri VM, Rubenfeld GD, Thompson BT (2012). Acute respiratory distress syndrome: the Berlin definition. JAMA.

[19] Pedersen SF, Ho YC (2020). SARS-CoV-2: a storm is raging (Epub ahead of print). J Clin Invest.

[20] Huang C, Wang Y, Li X (2020). Clinical features of patients infected with 2019 novel coronavirus in Wuhan, China. Lancet.

[21] Zhou Y, Fu B, Zhen X (2020). Pathogenic T cells and inflammatory monocyte incite inflammatory storm in severe COVID-19 patients. Natl Sci Rev.

[22] Li CK, Wu H, Yan H (2008). T cell responses to whole SARS coronavirus in humans. J Immunol.

[23] Mehta P, McAuley DF, Brown M, Sanchez E, Tattersall RS, Manson JJ (2020). COVID-19: consider cytokine storm syndromes and immunosuppression. Lancet.

[24] Liu Y, Sun W, Li J, Chen L, Wang Y, Zhang L, Yu L (2020). Clinical features and progression of acute respiratory distress syndrome in coronavirus disease 2019 (IN PRESS). medRxiv.

[25] Wang D, Hu B, Hu C (2020). Clinical characteristics of 138 hospitalized patients with 2019 novel coronavirus-infected pneumonia in Wuhan, China (Epub ahead of print). JAMA.

[26] Mukherjee A, Ahmad M, Frenia D (2020). A coronavirus disease 2019 (COVID-19) patient with multifocal pneumonia treated with hydroxychloroquine. Cureus.

[27] Holshue ML, DeBolt C, Lindquist S (2020). First case of 2019 novel coronavirus in the United States. N Engl J Med.

[28] Russell B, Moss C, George G, Santaolalla A, Cope A, Papa S, Van Hemelrijck M (2020). Associations between immune-suppressive and stimulating drugs and novel COVID-19—a systematic review of current evidence. Ecancermedicalscience.

[29] Zhao H, Shen D, Zhou H, Liu J, Chen S (2020). Guillain-Barré syndrome associated with SARS-CoV-2 infection: causality or coincidence?. Lancet Neurol.

[30] Poyiadji N, Shahin G, Noujaim D, Stone M, Patel S, Griffith B (2020). COVID-19-associated acute hemorrhagic necrotizing encephalopathy: CT and MRI features (Epub ahead of print). Radiology.

[31] Wang Y, Wang Y, Chen Y, Qin Q (2020). Unique epidemiological and clinical features of the emerging 2019 novel coronavirus pneumonia (COVID‐19) implicate special control measures (Epub ahead of print). J Med Virol.

[32] (2020). WHO: laboratory testing for 2019 novel coronavirus (2019-nCov) in suspected human cases. https://www.who.int/publications-detail/laboratory-testing-for-2019-novel-coronavirus-in-suspected-human-cases-20200117.

[33] Kanne JP, Little BP, Chung JH, Elicker BM, Ketai LH (2020). Essentials for radiologist on COVID- 19: an update-Radiology Scientific Expert Panel (Epub ahead of print). Radiology.

[34] Huang Y, Wang S, Liu Y (2020). A preliminary study on the ultrasonic manifestations of peripulmonary lesions of non-critical novel coronavirus pneumonia (COVID-19) (IN PRESS). Res Square.

[35] Yao X, Ye F, Zhang M (2020). In vitro antiviral activity and projection of optimized dosing design of hydroxychloroquine for the treatment of severe acute respiratory syndrome coronavirus 2 (SARS-CoV-2) (Epub ahead of print). Clin Infect Dis.

[36] Gautret P, Lagier JC, Parola P (2020). Hydroxychloroquine and azithromycin as a treatment of COVID-19: results of an open-label non-randomized clinical trial (Epub ahead of print). Int J Antimicrob Agents.

[37] Warren TK, Jordan R, Lo MK (2016). Therapeutic efficacy of the small molecule GS-5734 against Ebola virus in rhesus monkeys. Nature.

[38] Wang M, Cao R, Zhang L (2020). Remdesivir and chloroquine effectively inhibit the recently emerged novel coronavirus (2019-nCoV) in vitro. Cell Res.

[39] Chen J, Liu D, Liu L (2020). A pilot study of hydroxychloroquine in treatment of patients with common coronavirus disease-19 (COVID-19). Zhejiang Da Xue Xue Bao Yi Xue Ban.

[40] Cao B, Wang Y, Wen D (2020). A trial of lopinavir-ritonavir in adults hospitalized with severe COVID-19 (Epub ahead of print). N Engl J Med.

[41] Shanker A, Bhanu D, Alluri A, Gupta S (2020). Whole genome sequence analysis and homology modelling of a 3C like peptidase and a non-structural protein 3 of the SARS-CoV-2 shows protein ligand interaction with an aza-peptide and a noncovalent lead inhibitor with possible antiviral properties (IN PRESS). ChemRxiv.

[42] Kim D, Lee JY, Yang JS, Kim JW, Kim VN, Chang H (2020). The architecture of SARS-CoV-2 transcriptome (Epub ahead of print). Cell.

[43] Singhal T (2020). A review of coronavirus disease-2019 (COVID-19). Indian J Pediatr.

[44] Tang X, Wu C, Li X (2020). On the origin and continuing evolution of SARS-CoV-2 (Epub ahead of print). Natl Sci Rev.

[45] Tan L, Kang X, Zhang B (2020). A special case of COVID-19 with long duration of viral shedding for 49 days (IN PRESS). medRxiv.

[46] Chu H, Zhou J, Wong BH (2016). Middle East respiratory syndrome coronavirus efficiently infects human primary T lymphocytes and activates the extrinsic and intrinsic apoptosis pathways. J Infect Dis.

[47] Zheng HY, Zhang M, Yang CX (2020). Elevated exhaustion levels and reduced functional diversity of T cells in peripheral blood may predict severe progression in COVID-19 patients. Cell Mol Immunol.

[48] Ye G, Pan Z, Pan Y (2020). Clinical characteristics of severe acute respiratory syndrome coronavirus 2 reactivation. J Infect.

[49] Chen D, Xu W, Lei Z, Huang Z, Liu J, Gao Z, Peng L (2020). Recurrence of positive SARS-CoV-2 RNA in COVID- 19: a case report. Int J Infect Dis.

